# Research progress on miRNAs function in the interaction between human infectious viruses and hosts: A review

**DOI:** 10.17305/bb.2024.10821

**Published:** 2024-12-01

**Authors:** Xiaotong Wang, Wenchang Zhao

**Affiliations:** 1 Heilongjiang University of Traditional Chinese Medicine, Heilongjiang, China; 2 School of Pharmacy, Guangdong Medical University, Dongguan, China

**Keywords:** MicroRNAs (miRNAs), virus, virus–host interactions

## Abstract

MicroRNAs (miRNAs) represent a class of non-coding small RNAs that are prevalent in eukaryotes, typically comprising approximately 22 nucleotides and have the ability to post-transcriptionally regulate gene expression. miRNAs exhibit diverse types and functions, with mechanisms of action that include cell differentiation, proliferation, apoptosis, and regulation of signaling pathways. Both viruses and their hosts can encode miRNAs, which serve as crucial effector molecules in the complex interaction between viruses and host cells. Host miRNAs can either directly interact with the virus genome to inhibit virus replication or facilitate virus replication by providing necessary substances. Viral miRNAs can directly bind to host mRNAs, thereby influencing translation efficiency, suppressing the immune response, and ultimately enhancing virus replication. This article comprehensively reviews the roles of miRNAs in virus–host interactions, aiming to provide valuable insights into viral pathogenic mechanisms and potential therapeutic approaches.

## Introduction

MicroRNAs (miRNAs) are non-coding small RNAs prevalent in eukaryotes, typically comprising approximately 22 nucleotides, and they possess the capability to post-transcriptionally regulate over 60% of protein-coding genes [1]. miRNAs are widely prevalent across various organisms, displaying a diverse range of types and functions. They primarily function in regulating various aspects, including cell growth, differentiation, proliferation, apoptosis, and signaling pathways, while also contributing to the pathogenesis of diseases [2]. For instance, miR-92b-5p, miR-128-3p, and miR-26 have been shown to promote the differentiation of bone marrow mesenchymal stem cells [3–5]; miR-769-3p has been found to inhibit the proliferation and migration of nerve cells infected with Kaposi’s sarcoma-associated herpesvirus (KSHV) [6].

Viruses are non-cellular organisms composed of nucleic acids and proteins [7], which have the potential to cause diseases in both plants and animals. Virus replication is dependent on the host cell, with the key factor being the virus’s capability to circumvent the host’s immune response and exploit its resources for replication [8].

miRNAs encoded by Epstein–Barr virus (EBV) were first discovered by Pfeffer et al. in 2004 [9]. Further studies have subsequently confirmed the crucial role of these miRNAs in the viral infection process within hosts, thereby advancing research on viral miRNAs. Research has gradually revealed a close association between virally encoded miRNAs and viral infections [10]. Following viral infection, the host can directly impact the virus by regulating miRNA expression or through alternative inhibitory pathways [11]. Conversely, viruses modulate the expression of host miRNAs, enabling them to bind to the 3′ or 5′ UTRs of host mRNAs and subsequently affect translation efficiency and protein expression levels. This allows viruses to suppress the host’s immune response, evade immune clearance, and proliferate [12, 13]. Recent research indicates that viral miRNAs can influence viral survival through the regulation of cellular genes, offering new approaches for the treatment of viral diseases [14]. Hence, this article examines the relationship between viruses and miRNAs and explores their potential utility in antiviral therapy.

### miRNA synthesis pathway

#### Synthesis pathways of host miRNAs

**Figure 1.**
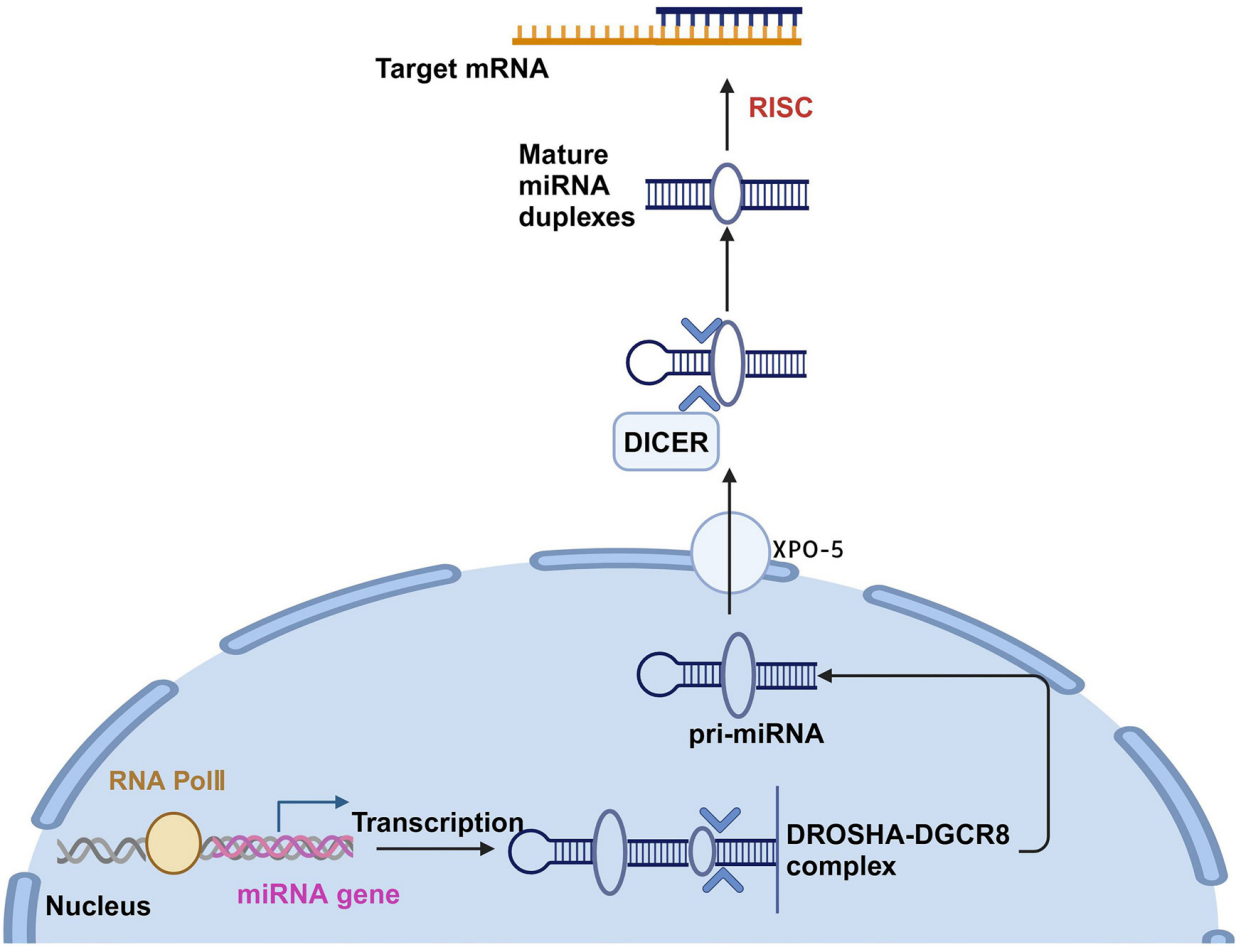
**miRNA pathway.** miRNAs are initially transcribed in the nucleus as pri-miRNAs. These pri-miRNAs are then processed by the Drosha–DGCR8 complex into precursor miRNAs (pre-miRNAs), which are subsequently exported to the cytoplasm via the XPO-5 transport system. Once in the cytoplasm, pre-miRNAs are further processed into mature miRNAs. These mature miRNAs play critical roles in regulating gene expression, primarily by facilitating the storage or degradation of mRNA transcripts. miRNAs: MicroRNAs; pri-miRNAs: Primary miRNAs; XPO-5: Exportin-5.

miRNAs play a crucial role in regulating cellular physiological and pathological processes. The biosynthesis of miRNAs involves intricate steps mediated by various key enzymes and other components. miRNA-encoding genes are first transcribed into primary miRNAs (pri-miRNAs). Pri-miRNAs are then cleaved into a shorter stem-loop intermediate, called pre-miRNA, by the combined action of RNA polymerase III (RNA pol III) and the Drosha-DGCR8 complex. Pre-miRNA molecules have distinct structural features crucial for miRNA functions, such as a 5′ phosphate group and a 3′ overhang. In the nucleus, Exportin 5 (XPO5) recognizes the 3′ end of pre-miRNA and, relying on Ran-GTP action, transports it to the cytoplasm [15]. In the cytoplasm, the Dicer enzyme cleaves pre-miRNA, producing mature double-stranded miRNAs of approximately 22 nucleotides in length. The mature miRNAs form a “double helix” structure with the complementary sequence of the target mRNA. This is followed by unwinding, allowing one strand to bind to the RNA-induced silencing complex (RISC). Through this process, miRNAs interact with the mRNA of target genes, thereby regulating gene expression [16]. Thus, miRNAs play a pivotal role in cellular gene expression regulation. The process of miRNA biogenesis is illustrated in Figure 1.

#### Viral miRNA synthesis pathways

The synthesis of miRNAs encoded by DNA viruses exhibits diversity. Certain miRNAs encoded by DNA viruses adhere to the conventional synthesis pathway, analogous to miRNA generation in eukaryotic cells. The synthesis of miRNAs involves the enzymes Drosha and Dicer. Drosha initially cleaves the pri-miRNA to form pre-miRNA, which is subsequently processed by Dicer to yield mature miRNAs. For instance, miR-3 encoded by the Hepatitis B virus (HBV) undergoes biosynthesis via a Drosha–Dicer-dependent mechanism [17]. Nevertheless, not all miRNAs encoded by DNA viruses adhere to this classical pathway. As an example, the synthesis of pri-miRNA encoded by Murine gamma-herpesvirus 68 (MHV68) diverges from the classical synthesis pathway [18]. These miRNAs are transcribed by RNA pol III promoters situated within adjacent viral transfer RNA (tRNA)-like sequences. These pri-miRNAs bypass processing by Drosha and are instead formed by cellular tRNase Z, primarily acting downstream of the tRNA structure to liberate the hairpin structure of pre-miRNA. These pre-miRNAs are then processed by Dicer, ultimately resulting in the generation of mature miRNAs.

**Figure 2.**
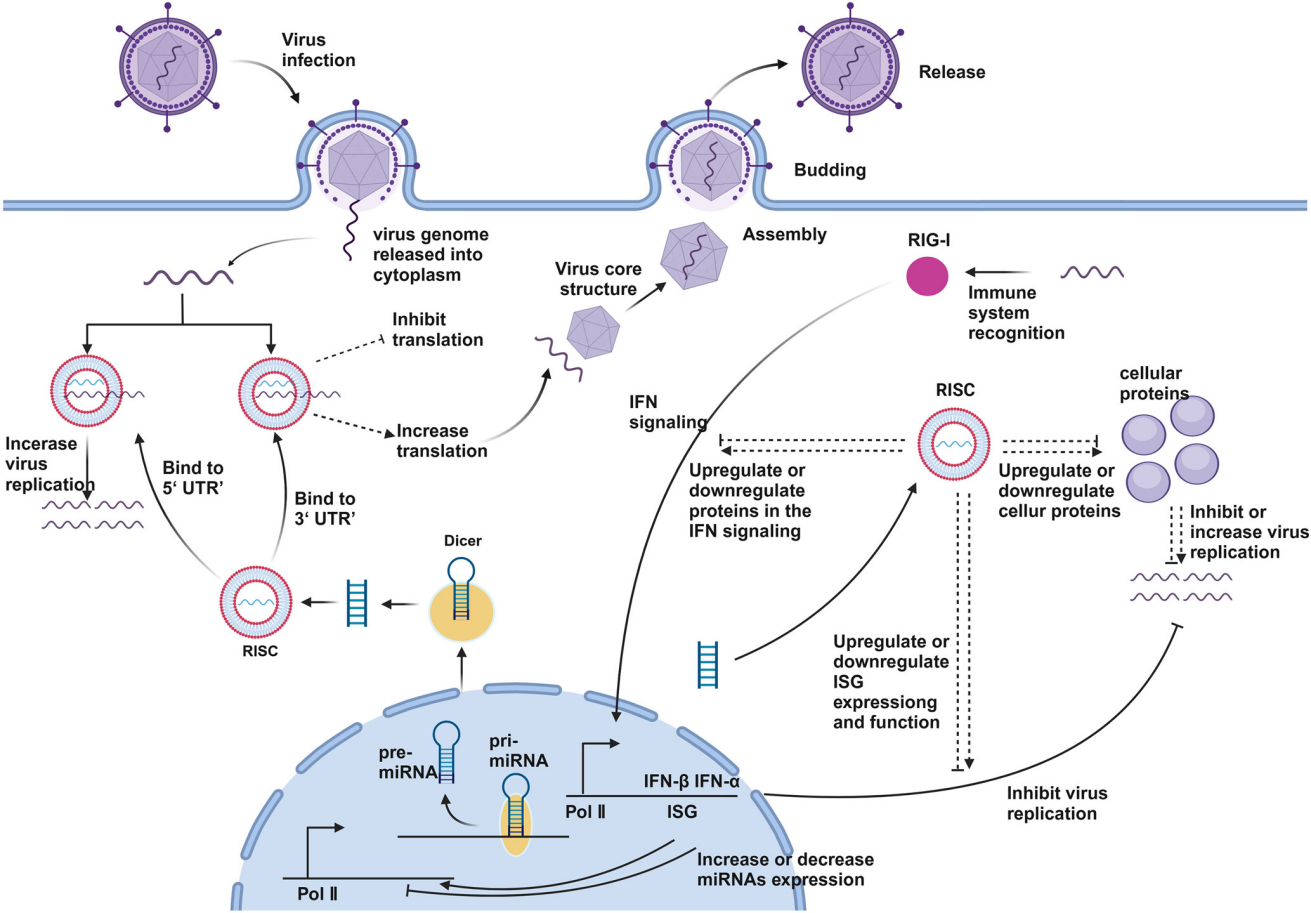
**The virus–host interaction.** Upon infecting the host cell, the viral genome is released into the cytoplasm. Within the host cell, miRNA precursors are processed by the enzyme Dicer to generate mature miRNAs, which then associate with the RISC complex. These mature miRNAs can bind to the viral genome’s 5’ UTR or 3’ UTR, resulting in either the enhancement or inhibition of translation, thereby influencing viral replication. Viral infection also activates the RIG-I pathway, leading to the secretion of interferons (IFN-α and IFN-β) and the regulation of interferon-stimulated genes (ISGs), which in turn affect miRNA expression levels. By modulating the expression of host and viral proteins, miRNAs ultimately either suppress or enhance viral replication, impacting the completion of the viral life cycle. RISC: RNA-induced silencing complex; miRNAs: MicroRNAs.

Both DNA and RNA viruses, including human immunodeficiency virus (HIV) and Rotavirus (RV), have the capability to encode miRNAs. miRNAs encoded by RNA viruses typically undergo nonclassical biosynthetic pathways, showcasing a diverse and complex synthesis process. Some RNA viruses encode miRNAs capable of bypassing the Drosha enzyme processing step and proceeding directly to Dicer enzyme processing. For instance, Adenovirus (Adv) pre-miRNA can circumvent the Drosha step [19]. In contrast, BHK cells infected with recombinant Sindbis viruses (rSINVs) are capable of expressing cytoplasmic-derived pri-miRNA, whose processing and maturation necessitate the infiltration of Drosha enzyme into the cytoplasm [20]. Furthermore, retroviruses, including Bovine leukemia virus (BLV) and primate herpesvirus saimiri (HVS), also exhibit comparable nonclassical miRNA synthesis traits. In BLV, miRNA synthesis manifests through primary transcription products generated by RNA polymerase III (pol III), a process that demands Dicer enzyme but not Drosha enzyme [21]. Conversely, HVS expresses Sm-class U RNAs (HSUR) that are transcribed by RNA polymerase II (RNAP II). These RNAs undergo processing by the integration complex to yield pre-miRNA, subsequently entering the classical biosynthetic pathway where they are processed by Dicer to produce mature miRNAs [22].

The diversity of viral miRNA synthesis is revealed by these discoveries, which enhance our understanding of virus adaptation to and influence on host cells through the regulation of host gene expression. Furthermore, a new perspective for studying the complex virus–host interactions is provided by these findings, potentially offering clues to the development of novel therapeutic strategies targeting these viruses.

### Patterns of virus–host interactions

The expression levels of target genes can be influenced to a certain extent by both viral and host miRNAs. Many processes related to viral infection, such as viral entry, latency, reactivation, immune response, and other biological processes, involve miRNAs. For example, lysis can be controlled by viral miRNAs, allowing the virus to enter a latent state and thus evade recognition by host cells [22]. Apoptosis in host cells can also be suppressed during viral proliferation to promote massive viral replication [23]. Immune evasion can be achieved by viral miRNAs by reducing the levels of specific proteins [24, 25]. On the other hand, viruses can be recognized by host-encoded miRNAs, prompting immune responses in host cells to eliminate them [26]. For instance, the activation of STAT1 to mount an antiviral response is promoted by miR-155-5p [27]. Moreover, virus replication may also be promoted by the interaction between host miRNAs and viruses [11]. Binding sites on the 3’ or 5’ UTRs of mRNAs interact with miRNAs, which can alter mRNA stability or inhibit its translation [12, 28].

As observed, miRNAs perform various functions in the intricate interplay between viruses and their hosts, from regulating the virus lifecycle to determining the survival of host cells. This bidirectional regulatory mechanism not only underscores the importance of miRNAs in viral infection processes but also indicates their potential utility in disease prevention and therapy [29]. The dynamic interaction between viruses and their hosts is shown in Figure 2. In subsequent sections, we will illustrate the use of miRNAs in the relationship between viruses and their hosts through various examples.

**Table 1:** Host miRNAs and their functions

**Virus type**	**Virus**	**miRNA**	**Function and mechanism**	**Ref.**
ssRNA	HIV	miR-17	Inhibits expression, aids replication	30
	ZIKV	miR-103a-3	Activates p38 MAPK, aids replication	31
		miR-142-5p	Boosts interferon responses, suppresses replication	51
		miR-16-5p	Promotes apoptosis, inhibits replication	56
	CVB3	miR-107	Upregulates Wnt/β-catenin, increases capsid protein	32
	SeV	miR-1225-3p	Inhibits interferon production, aids replication	33
		miR-4661	Targets IFN-α mRNA, suppresses replication	54
	HCV	miR-122	Circumvents immunity, promotes replication	34
		miR-27	Increases triglycerides, facilitates entry	36
	RSV	miR-29a	Decreases IFNAR1, facilitates replication	37
		miR-24, miR-124a, miR-744	Inhibits p38 MAPK, suppresses replication	58
	JEV	miR-432	Suppresses NF-κB, evades immune responses	38
		miR-499-5p	Downregulates P50, decreases IL-6 and IL-8	39
	VSV	miR-24, miR-93	Reduces mRNA levels, increases susceptibility	43
	SARS-CoV-2	miR-148a, miR-590	Suppresses USP33 and IRF9, inhibits pathways	57
	BoDV-1	miR-505	Reduces HMGB1, inhibits autophagy and replication	60
	EV71	miR-124-3p	Downregulates STAT3, suppresses replication	59
		miR-296-5p	Inhibits VP1 and VP3, suppresses replication	47
	IAV	miR-141, miR-324-5p	Regulate antiviral genes, inhibit replication	55
dsRNA	RV	miR-4301	Influences glycogen synthase, promotes replication	35
		miR-7, miR-125b-1-3p	Modulate transcripts, resist infection	44
dsDNA	HPV	miR-145	Targets E1 and E2, suppresses amplification	48
	HBV	miR-1231	Reduces core protein, inhibits replication	49
	HCMV	miR-200	Targets UL122, reduces viral titer	50
		miR-182	Targets FoxO3, activates IFN-1, suppresses replication	66
	HSV-1	miR-138	Represses lytic genes, promotes latency	40
		miR-183, miR-96, miR-182	Inhibit FoxO, interfere with regulation	41
	HSV	miR-24	Binds STING mRNA, promotes replication	67
	KSHV	pre-miR-K1	Suppresses KSHV miRNAs	63
		miR-127-3p	Enhances transcription, activates E2F and Myc	64
		miR-93	Activates IFN-JAK-STAT, enhances immune response	65

miRNAs encoded by host cells significantly impact the viral replication process by regulating their own expression levels and manipulating cellular functions to promote viral replication. Viruses can affect the expression of miRNAs by binding to regulatory sequences of host miRNAs, thereby intervening in the host’s immune response and cellular functions, including immune evasion. Additionally, host miRNAs can directly or indirectly suppress viral gene expression and enhance the host’s antiviral capabilities by activating specific signaling pathways. miRNAs: MicroRNAs; HIV: Human immunodeficiency virus; ZIKV: Zika virus; CVB3: Coxsackievirus B3; SeV: Sendai virus; HCV: Hepatitis C virus; RSV: Respiratory syncytial virus; JEV: Japanese Encephalitis Virus; VSV: Vesicular stomatitis virus; SARS-CoV-2: Syndrome-associated coronavirus2; BoDV-1: Bornavirus; EV71: Enterovirus 71; IAV: Influenza A Virus; RV: Rotavirus; HPV: Human papillomavirus; HBV: Hepatitis B virus; HCMV: Human cytomegalovirus; HSV-1: Herpes simplex virus 1; KSHV: Kaposi’s sarcoma-associated herpesvirus.

#### The host encodes miRNAs to act on the host or virus

Host miRNAs can participate in the entire process of virus replication and also exert antiviral effects in various ways. The summary of host miRNA and its related functions is shown in Table 1.

*miRNAs are involved in viral replication*

When a virus contacts a host cell, it binds to specific receptors on the cell membrane, activating intracellular signaling pathways either through these receptors or by entering the cell. This activation stimulates miRNA production within the cell, creating an environment that supports viral proliferation [11]. The interaction between the virus and the host cell is crucial for the viral lifecycle and the host’s immune response. Host cell-encoded miRNAs facilitate viral replication by modulating the expression of intracellular target genes. Viral products can influence miRNA levels by binding to regulatory sequences upstream of host miRNA genes. The miRNAs then bind to the 5′ UTR of target transcripts, regulating protein synthesis. These proteins can either aid in viral replication or suppress host antiviral responses, promoting immune evasion. Changes in host miRNA levels can enhance viral gene expression and transcription, increasing replication capacity or prolonging latency.

*miRNA-mediated enhancement of viral replication:* Several miRNAs have been identified to enhance viral replication through various mechanisms. In the case of HIV, the virus relies on the histone acetyltransferase Tat cofactor PCAF to inhibit the expression of miR-17, which is beneficial for its replication [30]. Zika Virus (ZIKV) exploits miR-103a-3, which targets OTUD38 to activate the p38 MAPK signaling pathway, facilitating ZIKV replication [31]. Similarly, Coxsackievirus B3 (CVB3) infection induces miR-107, which upregulates proteins associated with the Wnt/β-catenin signaling pathway, subsequently elevating the expression of the viral capsid protein, thus facilitating viral replication [32]. Sendai Virus (SeV) increases the expression of miR-1225-3p, promoting viral replication by inhibiting interferon production [33]. Hepatitis C virus (HCV) utilizes miR-122 to circumvent host cell immunity, creating a favorable environment for its proliferation and persistent replication within host cells [34]. Additionally, miR-4301 has been found to promote RV replication through its influence on glycogen synthase during the infection process [35].

*miRNA-mediated facilitation of viral entry:* In addition to enhancing viral replication, certain miRNAs also facilitate viral entry into host cells. For instance, HCV employs miR-27, which elevates triglyceride levels through the inhibition of peroxisome proliferator-activated receptor α (PPARα). This increase in triglycerides facilitates HCV’s entry into host cells and subsequent infection [36].

*miRNA-mediated evasion of host immune responses:* Viruses also exploit miRNAs to evade host immune responses. Respiratory Syncytial Virus (RSV) utilizes its NS1 nonstructural protein to induce the upregulation of host miR-29a. This upregulation leads to decreased expression of IFNAR1, facilitating RSV replication [37]. Japanese Encephalitis Virus (JEV) manipulates miR-432 to suppress NF-κB activity and disrupt the Jak-STAT signaling pathway, aiding in the virus’s evasion of cellular immune responses [38]. Additionally, JEV induces miR-499-5, which downregulates P50 expression, leading to decreased levels of IL-6 and IL-8, thereby aiding the virus in evading host clearance mechanisms [39]. Herpes Simplex Virus 1 (HSV-1) uses miR-138 to repress lytic cycle genes by targeting ICP0, Oct-1, and Foxc1, promoting epigenetic gene silencing and creating favorable conditions for latent infection [40]. The combined inhibitory effect of miR-183, miR-96, and miR-182 on FoxO protein family members further suggests HSV-1’s ability to interfere with host miRNA regulation, adding complexity to the regulation of viral replication [41].

*miRNA involvement in immune regulation:* Certain miRNAs are involved in immune regulation, which viruses can exploit to their advantage. ZIKV infection and persistence have been shown to correlate with miRNAs involved in immune regulation within the placental microenvironment [42], suggesting a complex interaction between the virus and host immune responses. In the case of vesicular stomatitis virus (VSV), the knockout of Dicer1 in mice leads to decreased expression of miR-24 and miR-93. This reduction in miRNA levels subsequently lowers VSV mRNA levels and increases the susceptibility of mice to VSV infection, highlighting the role of these miRNAs in immune regulation [43]. African green monkey kidney (Vero) cells, known as MA104, exhibit resistance to RV infection by upregulating the expression of miR-7 and miR-125b-1-3p, which modulate relevant transcripts [44].

*Antiviral effects of miRNAs*

Host miRNAs play a crucial role in antiviral defense by targeting viruses directly or indirectly through diverse mechanisms, thereby inhibiting their replication. They can bind to the 3′ UTR of RNA virus genomes, effectively suppressing the translation of viral genes and decreasing viral protein production [45]. Upon viral infection, host miRNAs not only activate the immune system but also regulate interferon production and ISG expression, essential for suppressing viral replication and transmission [46]. Host miRNA defense strategies include directly targeting viral genes to suppress their expression and interrupting viral entry into cells to mitigate infection. Furthermore, host miRNAs can modulate intracellular signaling pathways, such as interferon, MAPK, and NF-κB, significantly influencing the viral lifecycle and pathogenesis. By regulating gene expression directly and indirectly, miRNAs suppress viral replication, activate immune responses, and facilitate the elimination of infected cells or viruses through apoptosis, thus enhancing the host’s antiviral defense mechanisms.

*Direct suppression of viral gene expression:* Host miRNAs can directly target viral genes, suppressing their expression and consequently reducing viral replication.

*Direct targeting of viral genes:* miR-296-5p inhibits the synthesis of viral proteins VP1 and VP3 in Enterovirus 71 (EV71) infection, thus suppressing viral genome replication [47]. Similarly, miR-145 targets the E1 and E2 open reading frames of human papillomavirus (HPV), suppressing viral genome amplification and release [48]. miR-1231, exhibiting high homology with HBV mRNA, reduces the expression of the HBV core protein, thereby inhibiting HBV replication [49]. miR-32 directly resists human foamy viruses (PFV) by recognizing and binding to viral genes [11]. miR-200 targets the 3’UTR of the viral gene UL122 in human cytomegalovirus (HCMV), inhibiting viral translation and reducing viral titer [50].

*Modulation of host immune responses:* Host miRNAs also regulate immune responses, which are crucial for suppressing viral replication and transmission.

1. *Enhancement of interferon responses:* During Influenza A Virus (IAV) infection, miR-142-5p and the PI3K/AKT signaling pathway influence DNMT1 expression, which boosts RIG-I-mediated interferon responses, ultimately suppressing IAV replication [51, 52]. miR-155 regulates the expression of type I interferon and interferon-stimulated genes, thereby suppressing ZIKV replication [53]. miR-4661 targets the 3′ end of IFN-α mRNA in macrophages infected with SeV, suppressing SeV replication [54].
2. *Activation of antiviral signaling pathways:* miR-141 and miR-324-5p regulate antiviral genes induced by IFN and IL-6 signaling, inhibiting IAV replication [55]. By activating NF-κB and increasing the expression of the antiviral gene CXCL10, miR-16-5p promotes apoptosis, thereby inhibiting ZIKV replication [56]. miR-148a and miR-590 suppress USP33 and IRF9 expression in syndrome-associated coronavirus2 (SARS-CoV-2) infection, inhibiting the TNF-α, NF-κB, and IFN-β pathways and exerting antiviral effects [57].

*Regulation of intracellular signaling pathways:* Host miRNAs modulate intracellular signaling pathways, significantly influencing the viral lifecycle and the pathogenesis of viral infections.

1. *Pathway modulation:* miR-24, miR-124a, and miR-744 jointly target the MAPK14 gene, suppressing the expression and activation of p38 MAPK, thereby inhibiting RSV replication [58].
2. *Suppression of host genes for viral benefit:* miR-124-3p inhibits EV71 replication by downregulating STAT3, P-STAT3, CCND2, and MMP2 proteins, thus suppressing the transcriptional activity of STAT [59]. miR-505 reduces HMGB1 expression, inhibiting autophagy in host cells and suppressing Bornavirus (BoDV-1) replication [60].
3. *Regulation of cell growth and apoptosis pathways:* miR-125b-5p regulates multiple genes involved in cell signaling pathways linked to cell growth and apoptosis, thereby inhibiting JEV replication [61].

*miRNA-mediated inhibition of viral entry:* Host miRNAs can interrupt viral entry into cells to mitigate infection. Inhibition of viral entry: The insertion of miR-205 into the CVB3 viral genome exhibits dual effects: suppressing viral replication and enhancing the ability to kill host cells [62].

*Modulation of viral replication through host miRNA regulation:* Host miRNAs can also regulate the replication of viruses by modulating the expression of host genes that viruses rely on.

1. *Suppression of viral miRNAs:* The expression of the entire set of miRNAs encoded by KSHV can be suppressed by pre-miR-K1 [63]. By suppressing the expression of SKP2, miR-127-3p enhances the transcriptional activity of KSHV, leading to an increase in p21CIP1 expression and a decrease in the expression of cyclin E, cyclin A, and CDK2, thereby activating the transcriptional activity of E2F and Myc [64]. Meanwhile, the expression of host miR-93 is reduced by KSHV, activating the IFN-JAK-STAT pathway and enhancing the initiation of the host’s innate immune response, exerting an antiviral effect [65].
2. *Suppression of apoptosis:* miR-182 targets FoxO3 to activate IFN-1, suppressing HCMV replication [66]. miR-24, activated by kinases, binds to the 3’ UTR of STING mRNA, suppressing the host’s immune response and promoting enhanced replication of HSV [67].

#### The miRNAs encoded by the virus act on the virus itself or the host

Viral miRNAs regulate various intracellular processes, including signaling, immune response, autophagy, and apoptosis, by specifically targeting mRNAs within cells. They can bind to the 3′ or 5′ UTRs of both host and viral mRNAs, exhibiting diverse mechanisms of action. Binding to the 3′ UTR disrupts transcript stability and initiates mRNA degradation. Conversely, binding to the 5′ UTR can inhibit translation, enhance mRNA stability, prevent degradation, and upregulate gene expression [68]. Viral miRNAs can also mimic endogenous miRNAs or target unique binding sequences, forming a complex regulatory network during viral infection. This network influences numerous cellular functions, including suppressing viral replication, reducing viral toxicity, maintaining viral latency, and facilitating long-term coexistence and persistent infection within host cells. The interaction between viral miRNAs and host cell genes enhances viral replication by manipulating host messenger RNA expression. The summary of miRNAs encoded by viruses and their related functions is shown in Table 2.

**Table 2:** Viral miRNAs and their functions

**Virus type**	**Virus**	**miRNA**	**Function and mechanism**	**Ref.**
dsDNA	EBV	miR-2-5p	Suppresses IL-1 receptor	69
		miR-BART20-5p	Inhibits lytic cycle	71
		miR-BART6-5p, miR-BART18-5p, miR-BART11-5p, miR-BART2-5p	Suppresses replication, maintains latency	74, 75
	HCMV	miR-UL112	Disrupts interferon signaling	70
		miR-UL112, miR-UL148D, miR-UL22A, miR-UL36	Affects replication and infection processes	73
		miR-US25-1-5p, miR-US4-5p	Suppresses DNA synthesis and inflammation	78
		miR-UL112-3p	Aids immune evasion and latency	79
		miR-70-3p	Suppresses apoptosis	83
	KSHV	miR-K12-11	Modulates RTA expression	72
		miR-K9	Reduces DNA damage and apoptosis	84
		miR-K12-1-5p	Exacerbates apoptosis, weakens antiviral effects	85
	HSV	miR-H1-3p, miR-H6-5p	Restricts replication	76
		miR-24	Promotes replication	67
	HSV-1	miRNAs	Target lifecycle and immune genes	81
	SV40	miRNAs	Suppresses T antigen expression	80
	HHV-6A	miR-aU14	Enhances replication	86
	HBV	miR-3	Decreases apoptosis, enhances proliferation	82
ssRNA	WNV	Kun-miR-1	Facilitates replication	77

Viral miRNAs regulate the expression of host cell genes, both suppressing viral replication or reducing virulence to enter a latent phase for long-term coexistence with the host, and promoting viral replication by binding to specific binding sites. miRNAs: MicroRNAs; EBV: Epstein–Barr virus; HCMV: Human cytomegalovirus; KSHV: Kaposi’s sarcoma-associated herpesvirus; HSV-1: Herpes simplex virus 1; HHV-6A: Human herpesvirus 6A; SV40: Simian vacuolating virus 40; HBV: Hepatitis B virus; WNV: West nile virus.

*Suppression of host immune responses*

Viral miRNAs can directly target host immune genes to suppress immune responses, thereby aiding the virus in maintaining persistence and replication within the host. For instance, miR-2-5p, encoded by EBV, targets the 3′ UTR of the IL-1 receptor, suppressing its expression and enabling the virus to evade the host’s immune system [69]. HCMV encodes miR-UL112, which disrupts the innate immune response by suppressing type I interferon signaling [70].

*Maintenance of viral latency*

Viral miRNAs play a pivotal role in maintaining viral latency by targeting both viral and host genes to suppress replication and avoid immune detection. EBV-encoded miR-BART20-5p, for example, inhibits the lytic cycle by downregulating the expression of BZLF1 and BRLF1 genes, maintaining the virus in a latent state [71]. KSHV produces miR-K12-11, which modulates the expression of RTA and its downstream genes by targeting MYB, aiding in maintaining latent infection [72].

*Modulation of viral replication*

Viral miRNAs can directly influence viral replication processes by targeting viral or host factors involved in the replication cycle. HCMV encodes several miRNAs, including miR-UL112, miR-UL148D, miR-UL22A, and miR-UL36, all of which play roles in the replication, expression, and infection processes of the virus [73]. EBV also encodes miRNAs such as miR-BART6-5p, miR-BART18-5p, miR-BART11-5p, and miR-BART2-5p, which suppress viral replication and maintain latency by specifically targeting genes involved in the replication process [74, 75]. Moreover, HSV produces miR-H1-3p and miR-H6-5p, which complement each other to restrict or control viral replication, ensuring long-term infection in the host [76]. West Nile virus (WNV) encodes Kun-miR-1, which binds to GATA-4 mRNA, leading to the loss of GATA-4 function and promoting viral replication [77]. Lastly, HCMV-encoded miRNAs, such as miR-US25-1-5p and miR-US4-5p enable long-term coexistence in host cells by suppressing DNA synthesis, transcription, and the production of inflammatory mediators [78].

*Evasion of host immune recognition*

Viral miRNAs help evade host immune recognition by targeting key components of the immune response. HCMV produces miR-UL112-3p, which targets multiple viral transcripts, such as IE72, crucial for immune evasion and leading to latent infection [79]. Simian vacuolating virus 40 (SV40) encodes miRNAs that cleave their own mRNA, suppressing the expression of T antigens and aiding in evasion from host immune cell recognition and clearance [80]. HSV-1-encoded miRNAs primarily target viral genes involved in lifecycle transitions and regulate host genes related to antiviral immunity, contributing to the evasion and lifelong persistence of HSV-1 in the host [81].

*Regulation of host cell apoptosis and proliferation*

Viral miRNAs can modulate host cell apoptosis and proliferation pathways to enhance viral survival and replication. HBV encodes miR-3, which binds to the 3’ UTR of PTEN mRNA, resulting in downregulation of PTEN protein expression, decreased cell apoptosis, and enhanced cell invasion and proliferation capabilities [82]. MiR-70-3p, encoded by HCMV, suppresses cell apoptosis by downregulating the expression of the pro-apoptotic gene MOAP1 [83]. Additionally, KSHV encodes miR-K9, which reduces DNA damage and cell apoptosis induced by viral infection through the downregulation of caspase-3 and caspase-7 expression [84]. Furthermore, the miR-K12-1-5p encoded by KSHV exacerbates host cell apoptosis and leads to loss of cell viability, while weakening the antiviral effect of IFN β. [85]. Human herpesvirus 6A (HHV-6A) produces miR-aU14, which disrupts the mitochondrial structure of host cells, thereby enhancing replication [86].

### miRNAs diagnosis and treatment against viral pathogens

#### miRNAs diagnostics

In terms of diagnosis, the expression pattern of miRNAs exhibits high tissue specificity and disease relevance, rendering them potential molecular markers. Through the utilization of real-time quantitative PCR technology, the expression levels of miRNAs in various tissues can be precisely detected, thus providing compelling evidence for the early diagnosis of diseases. The stable presence of miRNAs in extracellular vesicles and other bodily fluids qualifies them as ideal candidates for noninvasive diagnosis, offering the potential to mitigate patient discomfort and diagnostic risks [87]. The expression levels of miRNAs can reflect disease progression and treatment efficacy in real time, thereby providing a foundation for physicians to adjust treatment plans. Consequently, the utilization of miRNAs for diagnostic purposes is anticipated to broaden the diagnostic scope and enhance the diagnostic accuracy of diverse pathologies.

Following infection with occult HBV (OBI), the serum expression levels of miR-122 and miR-130a miRNAs exhibited significant statistical differences compared to normal serum. This specific combination of miRNAs can serve as an accurate tool for detecting OBI [88]. MiR-340-3p and miR-451a function as biomarkers for HBV infection due to their role in the inhibition of ATF2 and HBV replication [89]. Compared to healthy individuals, patients with EV71 demonstrated notable differences in the expression levels of miRNAs, specifically miR-148a, miR-143, miR-324-3p, miR-545, and miR-140-5p. These miRNAs can facilitate the diagnosis and aid in the classification [90]. The expression levels of miR-134, miR-320c, miR-198, and miR-483-5p are elevated in the serum of HCV patients across various genotypes and individuals, potentially serving as markers for hepatitis C [91]. MiR-2392 is exclusively present in COVID-19-positive patients, offering novel evidence for the diagnosis of the disease [92]. Meanwhile, miR-155 demonstrates distinct expression patterns in SARS-CoV-2 and COVID-19, thereby serving as a valuable diagnostic and prognostic indicator [93]. Following HIV infection, the downregulation of miR-192-5p and miR-534 expression levels in patients’ eyes aids in determining the disease status of HIV-infected individuals [94].

#### miRNA therapy

In terms of therapeutic applications, miRNAs are capable of regulating the expression of numerous genes, and therapies utilizing miRNAs exhibit high efficiency and comprehensiveness. The specific binding of miRNAs to their target genes mitigates toxic side effects on normal cells during therapy, thereby enhancing treatment safety [95]. By modulating miRNA expression, the progression of various diseases can potentially be reversed. Integrating miRNA-based therapies with other treatment modalities can enhance therapeutic efficacy and reduce the likelihood of recurrence. As a novel drug target, miRNAs offer a fresh avenue for the development of innovative pharmacologic agents [96]. The design and development of drugs targeting specific miRNAs are anticipated to usher in revolutionary advancements in disease treatment.

Through RT-PCR analysis, Song et al. [97] observed disparities in the levels of miR-31, miR-29a, and miR-148a in peripheral blood mononuclear cells between IAV patients and healthy individuals. The utilization of inhibitors targeting these miRNAs has the potential to mitigate the toxicity associated with IAV. Studies have indicated an association between miR-155 and both viral load and cell proliferation in HBV. Inhibition of miR-155 has been shown to ameliorate HBV-induced pathologies [98]. According to Lin et al. [99] miR-548c-3p functions as a crucial negative regulator of TRIM22 in HBV patients undergoing interferon therapy. This discovery pertains to the role of interferonα. Assessing therapeutic efficacy reveals novel biomarkers and therapeutic targets, potentially leading to clinical breakthroughs. Miravirsen, the first anti-miRNA drug targeting HCV, is currently in Phase II clinical trials [100]. Clinical trials have shown that Miravirsen, an antisense oligonucleotide targeting miR-122, lowers HCV mRNA levels by prolonging its duration of action. This innovative medication potentially offers new hope for the treatment of individuals with HCV.

## Discussion

Viruses constitute a unique type of organism that depends on host cells for their survival and reproduction. To achieve their replication and ensure their persistence, viruses must influence cellular processes by regulating the host’s miRNA expression profile.

When viruses invade host cells, they disrupt the normal expression of miRNAs, resulting in dysregulation of the miRNA expression profile. On the one hand, viruses can employ their encoded proteins to directly target the host cell’s miRNA processing machinery, disrupting its normal function. On the other hand, viruses can indirectly influence the expression level of miRNAs by modulating transcription factors and epigenetic modifications in host cells. The imbalance in the miRNA expression profile further induces alterations in cellular processes, which are beneficial for virus replication and persistence. These alterations may encompass disruptions in cell cycle regulation, suppression of immune responses, and delayed cell apoptosis.

Numerous studies have established a correlation between virus-induced dysregulation of host miRNA expression profiles and alterations in cellular processes. For instance, specific viruses can elevate the expression levels of particular miRNAs within host cells, thereby suppressing the host cell’s antiviral immune response and creating a conducive environment for virus replication. Furthermore, certain viruses can also reduce the expression of miRNAs associated with cell apoptosis, thus impeding the process of host cell apoptosis and facilitating persistent viral infection.

The regulatory function of miRNAs at the single-cell level during viral infection significantly affects host response and viral dynamics. Through the use of single-cell RNA sequencing and imaging techniques, the complexity and heterogeneity of miRNA regulation can be accurately delineated. Single-cell RNA sequencing technology enables the analysis of gene expression patterns in individual cells, thus serving as a potent tool for investigating the function of miRNAs in viral infection processes [101]. Changes in miRNA expression levels across different cells can be observed through this technology, thereby elucidating the function of miRNAs in virus replication, host immune response, and cell fate determination. Furthermore, imaging technology aids in visually observing the localization of miRNAs within cells and its interactions with other molecules, thereby enhancing our comprehension of miRNA functions.

To gain a comprehensive understanding of how miRNAs regulate the interaction between viruses and hosts, high-throughput sequencing and bioinformatics methods can be employed. High-throughput sequencing allows for the simultaneous detection of the expression levels of thousands of miRNAs, enabling the identification of those miRNAs playing a crucial role in virus infection. Bioinformatics analysis aids in identifying the target genes of these miRNAs, predicting their functions, and constructing miRNA regulatory networks. These analyses not only facilitate the understanding of the specific mechanisms of miRNAs in viral infection but also provide a theoretical basis for the development of new antiviral strategies [102].

Currently, miRNAs are considered key regulatory factors in the interaction between viruses and hosts. Despite the continuous discovery of new host- and virus-encoded miRNAs, their mechanisms in immune responses still need further exploration owing to the diversity and complexity of their target genes and functions. miRNAs research is still in its infancy, and numerous unknown sequences and functional gaps remain to be filled. A key challenge lies in breaking through existing technologies and methods, particularly in analyzing the impact of miRNA translation and specific combinations on the host immune response. Meanwhile, the potential of miRNAs as early diagnostic markers and therapeutic targets warrants exploration. With the deepening of scientific research, it is anticipated that the blind spots regarding miRNA’s role in the interaction between viruses and hosts will gradually be elucidated, paving the way for new breakthroughs in the prevention and treatment of virus-related diseases.
